# Editorial: Explainable, Trustworthy, and Responsible AI for the Financial Service Industry

**DOI:** 10.3389/frai.2022.902519

**Published:** 2022-05-05

**Authors:** Branka Hadji Misheva, Jochen Papenbrock

**Affiliations:** ^1^Zurich University of Applied Sciences, Winterthur, Zurich, Switzerland; ^2^NVIDIA GmbH, Würselen, Germany

**Keywords:** explainable AI, ethical AI, trustworthy AI, responsible AI, Computational Trustworthy AI

More than 60 years have passed since the first coordinated research on Artificial Intelligence (AI) began at MIT when John McCarthy and Marvin Minsky founded the AI Lab. Since then, AI has developed into a wide-ranging tool that enables us to fundamentally rethink how data is integrated, analyzed and used for decision making. Growing pool of research is providing evidence of the many advantages that AI can bring to the financial sector: offering a new approach to risk management and compliance, reducing the cost of operations, increasing financial inclusion, implementing hyper-personalization, as well as automating tasks making operations more efficient.

Yet, finance service providers are slow to fully adopt AI-based systems in day-to-day tasks, in part because of the large legacy IT environment which might not be accommodating for advanced analytics. Another highly relevant barrier for wider adoption of AI in the context of the financial sector is related with the concept of explainability. Namely, AI solutions are often referred to as “black boxes” because typically it is difficult to trace the steps the algorithms took to arrive at its solution. This lack of transparency and explainability is then a critical point for policymakers and regulators who strive to promote and validate models that are robust and remain relatively stable after deployment. For example, in the context of credit scoring, there is a regulatory need that decisions are fair and unbiased. Furthermore, the GDPR provides a right to explanation, enabling users to ask for an explanation as to the decision-making processes affecting them. Thus, the adoption of innovative technologies must be done in a responsible, trustworthy way, especially in the context of the financial sector which impacts the overall economy.

On top of this fundamental need for explainability, the financial sector faces increasingly sophisticated adversaries having the capabilities to execute large scale data breaches, data tempering and loss of confidential information. This similarly calls for robust and stable methods that can handle noise and persist in view of adversarial corruption of data.

In this context, this Research Topic aims to include original papers proposing innovative methodologies for global or local explanations as well as assessing fairness and robustness of AI-based systems applied to financial problem sets.

Looking specifically at the audience-dependent nature of explainability, the study by Hadji Misheva et al. explores how various stakeholders within the Swiss financial industry view explainability and provides an in-depth discussion as to the potential and limitation of current XAI techniques. Such a study provides a key contribution to the literature by bridging the gap that exists between explainable techniques as deployed in the literature and the needs of the industry.

An additional contribution to the research selection, the study by Gramespacher and Posth, focuses on employing explainable AI for the optimalization of the return target function looking at the exemplary use case for credit assessment. The authors specifically argue that in case of strongly asymmetric costs of inaccurate prediction, the accuracy metric should be substituted for an economic target function. Furthermore, the application and results discussed confirm a key benefit associated with the emergence of Fintech credit providers. Namely, the authors observe that the profit-maximizing models (typically employed by traditional financial intermediaries) tend to reject surprisingly many of the non-defaulting contracts, thus limiting access to the credit market for a significant portion of agents. This gap can be filled by fintech credit providers which, through the use of alternative data and advance methodologies, can operate at a lower cost and thus contribute to broader benefits for financial inclusion.

Yet another contribution to the research collection focuses on the application of self-play algorithms to finance problem sets. Specifically, a central research question that Posth et al. aim to answer is whether AI methodologies like self-play can be applied to financial markets. The extensive literature review conducted by the authors indicates that despite the significant important of AI for risk management, big data analysis, credit risk and fraud detection, the application of self-play algorithms to financial markets seems to be underexploited in terms of both academic and industry-related research. Namely, the usage of self-play algorithms in trading is highly challenging, requires large data sets, multiple simulations and scenarios. The paper significantly contributes the discussion on the practicality of deploying AI in trading and forecasting financial markets.

Szepannek and Lübke present different definitions of fairness in cedit scoringplus a fairness correction algorithm Furthermore, the idea of population stability is transferred into a new group unfairness index which allows quantifying and comparing the degree of group fairness of different scoring models. A simulation study has been set up which makes use of a corrected version of the well-known German credit data. The results of the study are quite promising: Up to some degree fairness corrections are possible without strong loss in predictive.

Hall et al. provide a mini review on key definitions and considerations for using ML within the United States lending context. While questions remain as to which methods will be most useful for ensuring compliance with regulatory requirements, variants of constrained models, Shapley values, and counterfactual explanations appear to be gaining some momentum in the broader lending community. As models become more sophisticated, proper model governance, and human review, and closer collaboration between legal, compliance, audit, risk, and data science functions will likely only increase in importance.

In their perspective paper, Fritz-Morgenthal et al. focus on the management of the model risk of productive models in banks and other financial institutions and share the following seven insights:

1. there need to be general principles, requirements, and tests with focus on models' respective purposes, influence on human lives, and business impact, and special tests will be necessary for more complex or even dynamic models.
2. expertise/approaches of classical risk management/governance need to be combined with those of data science and AI knowledge.
3. many aspects of AI governance, algorithmic auditing, and risk management of AI systems can be addressed with technology and computing platforms.
4. an entire industry is about to emerge in this area.
5. Explainability, interpretability, and transparency of models, data, and decision making will be key to even enable an appropriate possibility to manage remaining model risks (“Explaining Explainable AI”).
6. One aspect of the “Explainable AI” agenda is to enable the fairness of AI decision making or decision support from a societal perspective (linked to the ESG agenda).
7. Last, they propose that the final decision about which model should be used, which one needs to be reviewed, and which models should be discontinued, should always be made by a human being. This ensures that the responsibility resides with the respective human decision maker, but is also an important control for drift in self-learning models.

We expect tremendous momentum in this highly interesting and relevant area. More approaches, algorithms, tools, frameworks, and technologies will be added, and enterprise-scale production-level deployment will occur. In the process, existing MLOps frameworks will be extended to include tools for AI governance, assurance, compliance, security, observability, certification, testing, and inspection, using tools from the emerging field of Computational Trustworthy AI currently being developed in projects such as Gaiax FAIC. Entire platforms, ecosystems, and industries will emerge to comply with AI regulations and increase trust in AI, fostering even broader adoption of AI in business and research.

## Author Contributions

All authors listed have made a substantial, direct, and intellectual contribution to the work and approved it for publication.

## Conflict of Interest

JP was employed by NVIDIA GmbH, Würselen, Germany. The remaining author declares that the research was conducted in the absence of any commercial or financial relationships that could be construed as a potential conflict of interest.

## Publisher's Note

All claims expressed in this article are solely those of the authors and do not necessarily represent those of their affiliated organizations, or those of the publisher, the editors and the reviewers. Any product that may be evaluated in this article, or claim that may be made by its manufacturer, is not guaranteed or endorsed by the publisher.

